# Review on Panic Buying Behavior during Pandemics: Influencing Factors, Stockpiling, and Intervention Strategies

**DOI:** 10.3390/bs14030222

**Published:** 2024-03-09

**Authors:** Reza Jazemi, Sajede Farahani, Wilkistar Otieno, Jaejin Jang

**Affiliations:** Department of Industrial & Manufacturing Engineering, University of Wisconsin-Milwaukee, Milwaukee, WI 53211, USA; sajede@uwm.edu (S.F.); otieno@uwm.edu (W.O.);

**Keywords:** panic, panic buying, influencing factor, intervention strategy, stockpiling, Socio-Economic Framework of Panic (SEFP)

## Abstract

Panic buying poses significant challenges for individuals and societies. This paper provides a literature review on the process by which a pandemic crisis evolves into panic buying behavior. The review offers a comprehensive perspective on studies related to panic buying and mitigation efforts, categorizing them based on their contributions in three stages: factors influencing panic buying, the process of transforming panic into increased demand and stockpiling, and applicable intervention strategies to mitigate panic situations. The paper introduces the Socio-Economic Framework of Panic (SEFP) to illustrate the interaction between demand and supply during a panic. The review identifies a lack of quantitative models explicitly correlating influencing factors with panic and estimating panic demand. Additionally, it reveals that suggested intervention strategies often lack practical implementation guidelines. Using the SEFP, the importance of considering interventions at various stages is highlighted, ranging from controlling influencing factors and panic demands to overseeing stockpiling and supply-related activities. The paper also identifies research gaps in both qualitative and quantitative modeling, policymaking, and governance.

## 1. Introduction

The goal of this review paper is to introduce a Socio-Economic Framework of Panic (SEFP) and utilize it for a systematic examination of past research articles. This systematic review delineates research opportunities to quantify the impacts of panic buying and potential considerations for panic mitigation. Panic buying is an extreme social response to disasters, characterized by a surge in consumers' accumulation of necessities due to the fear of future supply shortages. This phenomenon has been observed in various instances, including the outbreak of Severe Acute Respiratory Syndrome (SARS) in China [1], the Japanese earthquake [2], the influenza outbreak (H5N1) [3], as well as the outbreaks of influenza (H7N9) and SARS [4]. Notably, the COVID-19 pandemic over the past four years has been one of the most significant global disasters, exerting a profound impact on the economy, businesses, people's social lives, habits [5,6], and even individuals' physical health [7]. Both the industry and academia have reported numerous studies investigating panic buying behavior, some of which are summarized by Billore and Anisimova in Ref. [8].

In a contributed paper, Nguyen and Ilan [9] used a Life Years Index to conclude that the costs of the pandemic, measured only for 2020 and excluding any predicted costs for 2021, far outweighed the average annual costs associated with all other disasters aggregated together and/or the summed costs of all other epidemics. The impact of the pandemic was particularly exacerbated by international population interactions through travel and migration. The effects of panic buying on the US economy could be symbolized by its impact on the sales trend of toilet paper and hand sanitizer. According to Georgia-Pacific data, the average American household uses about 409 rolls of toilet paper a year, and people will use about 40% more toilet paper than usual if they spend all their time at home. However, their sales in the first quarter of 2020 indicated that people stockpiled their toilet paper for about three months. The fact is that the total days of lockdowns for the entire year of 2020 were much less than a month [10]. Hand sanitizer sales jumped 600% in 2020. Gojo Industries added its second hygiene factory, expecting new hygiene habits to persist after the COVID-19 pandemic [11].

In the case of regional and rapid disasters, such as earthquakes and floods, shortages can typically be promptly addressed by tapping into the limited emergency stocks or inventories of unaffected areas. However, a pandemic, with its potential for prolonged duration and widespread impact, presents a challenge that cannot easily be overcome solely through logistical enhancements. 

Significant endeavors have been dedicated to unraveling the intricacies of panic buying and comprehending the factors shaping this phenomenon. Numerous studies have approached panic as a psychological response, focusing on pinpointing the contributing factors to such reactions [12,13,14]. Some researchers have also investigated how panic can disrupt the equilibrium between supply and demand [15,16,17]. Numerous studies have explored strategies to alleviate the impact of panic buying on both individuals' lives [18,19,20] and on businesses [21,22,23,24]. 

However, these discussions frequently lack a comprehensive perspective that considers the interactive effects of crises, panic buying behavior, demand–supply relationships, and precise qualitative or quantitative solution approaches, as noted in Refs. [25,26,27,28,29]. This emphasizes the necessity for more thorough discussions in the following areas:

- The relative impact of numerous factors on panic buying behavior, including the influence of neighbors' behavior, disaster-related news, and empty store shelves.
- The transformation process through which individuals' psychological distress from panic is converted into panic-driven demand.
- Alteration in demand for different product types by panic.
- Changes in social habits and demand patterns during and after the panic.
- Appropriate interventions and the areas of impact.

In this paper, a systemic approach was employed to review papers to enhance the understanding of the panic process and address the aforementioned concerns. Specifically, it does the following:

1. Introduces the Socio-Economic Framework of Panic (SEFP) to achieve a more systematic and comprehensive perspective on the occurrence and management of panic situations.
2. Categorizes publications related to panic based on the SEFP.
3. Summarizes the interventions proposed to mitigate the panic situation.
4. Identifies gaps in current studies, formulates a future research agenda, and proposes considerations for policymaking.

With these objectives in mind, this paper unfolds as follows: Section 2 and Section 3 elaborate on the search process and the SEFP. Section 4 offers an in-depth survey of research on panic buying phenomena. The studies are categorized into three subsections based on the SEFP: influencing factors on panic, the transition from panic to panic demand and stockpiling, and intervention strategies. Section 5 concludes the paper by summarizing the study's implications and presenting a future research agenda.

## 2. Search Process

The research team conducted a comprehensive literature review on panic and panic buying behavior from 2005 to 2023. Various databases were utilized, including Business Source Premier, Google Scholar, Science Direct, Emerald Insight, Scopus, and Web of Science. The keywords that were employed were derived from bibliometric surveys [27,28]. They encompassed terms such as panic, panic buying, compulsive buying, hoarding, shop raiding, stockpiling, panic demand, disaster to panic, panic intervention, panic management, and panic mitigation.

Review papers were incorporated to establish the structure of the survey, to ensure the inclusion of comprehensive ranges of relevant study areas, and to minimize the possibility of overlooking pertinent research findings. This review excludes the papers that solely examined sharply increased demand without addressing influencing factors and those that explored the reactions to panic other than stockpiling.

This review covers a similar time span as Refs. [8,30], encompassing the last two decades. Notably, due to the significant impact of COVID-19 on panic buying, a substantial volume of research has been published in the last four years alone [23,25].

## 3. The Socio-Economic Framework of Panic (SEFP)

Panic buying, a psychological response triggered by uncertainty during a crisis, has been extensively researched since 2005. This phenomenon is influenced by a range of internal and external factors that shape individuals’ cognitive and emotional states [31] and disrupts the equilibrium between supply and demand. Panic results from certain circumstances such as significant disturbances that hinder rational decision making, the recurrence of product shortages, governmental inadequacy in managing panic-inducing factors, businesses’ inability to compensate for shortages, and a lack of clear visibility into the crisis duration.

**Figure 1:**
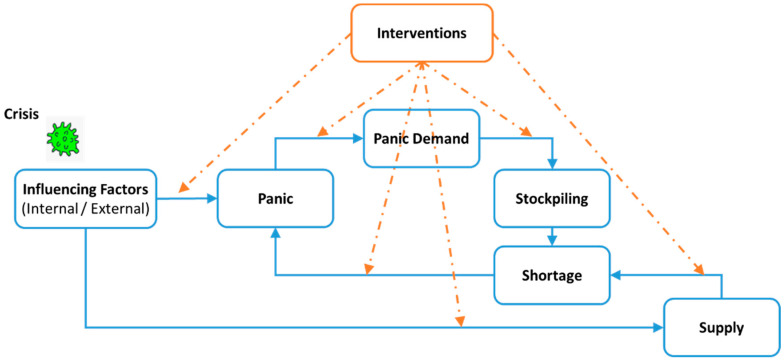
The Socio-Economic Framework of Panic (SEFP).

To obtain a comprehensive understanding of panic, scholars have recommended adopting a holistic and systematic approach [26]. The existing literature shows that consumers’ process of panic buying can be divided into four distinct stages: being informed, raising desire, intention to purchase, and decision to buy [14]. It has been observed that suppliers can effectively reduce panic buying by adeptly responding to customer demand [32,33,34]. Also, interventions at various stages of panic executed by stakeholders are essential to effectively manage and control panic buying behavior [22,23,35]. Considering the above-mentioned aspects, the Socio-Economic Framework of Panic (SEFP) was developed by expanding upon the stages of panic buying behavior proposed by Naeem, 2021 [14]. The SEFP offers a comprehensive perspective on the occurrence of panic and management of panic buying behavior, enabling both governmental and private entities to devise effective interventions. Figure 1 illustrates the SEFP model, including the interconnected influencing factors and their relationships involved in this phenomenon.

The SEFP consists of the following seven stages. The framework includes the following parameters:

- Influencing factors encompass a variety of internal and external factors that are impacted by crises, leading to a significant disturbance in people’s future expectations. Internal (IN) factors pertain to individuals’ personalities, feelings, and situation, and the external (EX) factors are related to the environment and society [36].
- Panic is an intense distressing emotional response caused by influencing factors, impairing people’s ability to think and react rationally, resulting in panic buying.
- Panic demand is people’s willingness to buy more than their usual demand to alleviate their intense fear.
- Stockpiling is individuals’ aggressive purchasing of goods to fulfill their panic demand, thereby disrupting the balance between demand and supply.
- Supply is an activity performed to respond to market demands.
- Shortage occurs when the supply is unable to meet the panic demand.
- Intervention is any preventive or corrective activity that brings the disrupted demand and supply to its balanced state.

The literature shows that researchers interchangeably use panic buying, compulsive buying, hoarding, and impulsive buying to address some or all of the seven stages in the SEFP. The widely referenced description of panic buying behavior in Ref. [37], given as “a phenomenon characterized by a sudden surge in excessive purchasing prompted by disasters or outbreaks, resulting in imbalances between supply and demand”, covers only three stages in the SEFP: influencing factor, stockpiling, and shortage. Others use the term panic buying to refer only to stockpiling [15,35,38]. The term compulsive buying is sometimes used when the buying results from panic [39], while this term is often used to show buying because of other negative feelings such as depression [17,40]. Also, the term hoarding is used to mean panic buying or, more often, the act of storing goods for future profit [41]. Similarly, impulsive buying is used to mean stockpiling [20,42].

## 4. Research Review on Panic Buying Behavior

The influencing factors of panic affect both the supply and demand sides. Much of the discussion on supply planning during pandemics can be found in the supply chain resilience literature [16,24,34,43,44,45]. However, most of the discussions have been about the efficiencies of product distributions. The factors and the process that affect panic demand have also been intensively discussed in the literature. Many of them qualitatively consider psychological aspects of people’s perception.

Papers on panic buying behavior were reviewed and discussed considering their area of focus in the SEFP. These studies are categorized into following three areas:

- Influencing factors of panic;
- Transformation of panic into panic demand and stockpiling;
- Interventions at each stage of the SEFP.

### 4.1. Influencing Factors of Panic

Panic can be caused by either internal (IN) or external (EX) factors. Some researchers have also grouped the factors into direct and indirect effects [25,46] and by rational or irrational motives [47].

The IN/EX classification offers better advantages than other groupings when devising appropriate intervention strategies; for IN factors, interventions focus on individuals, and for EX factors, they improve the environment. By differentiating subjective experiences from objective realities, the IN/EX classification clarifies the interconnectivity of individual perceptions and environmental factors. The direct/indirect classification becomes less significant when determining intervention strategies. The rational/irrational classification falls short for precise interventions, as rational factors require a response rather than an intervention and irrational factors are just the individuals’ mistakes that should be corrected. So, the influencing factors were separated into the following two groups. In the literature, it is observed that people use different terms to mean the same aspects of factors, as also shown below.

1. Internal factors:
  - Cognitive response: attitude, cues to action, self-efficacy, affective response on individuals, anticipated regret.
  - Perceived scarcity: fear of future unavailability, background rate, outcome expectation.
  - Perceived severity: intolerability, perceived lack of control.
  - Perceived susceptibility: fear of illness, fear of being affected.
  - Anxiety: bad mood, isolation, distrust.
  - Displacement.
2. External factors:
  - Information intensity: shared information, information availability, social media usage, eWOM (electronic Word-of-Mouth), rumors, information quality, effective spread of information and news.
  - Community preparedness: conformity of community, normative social influence, social norms, emotional contagion, social trust, demographics.
  - Neighboring effect: peer behavior, herd psychology, social influence, social inclination to buy more, observational learning, subjective norms.
  - Shortage: supply disruption, sufficiency of supplies, delivery limitation, limited quantity scarcity, empty shelves, food at hand.
  - Statistics: death/injury/infection rates, property damages.
  - Regulation: governmental intervention, lockdowns, rationing, price changes.

The literature has reported some contradictory findings. For instance, while Chua et al. [46] suggested that perceived severity indirectly affects perceived scarcity, Yuen et al. [48] argued against this relationship. Although Tan et al. [49] and Lehberger et al. [50] both utilized the elements of the Theory of Planned Behavior (TPB) to examine their effects on panic, their observations diverged. Specifically, Tan et al. [49] asserted that perceived behavioral control does not significantly affect panic, whereas Lehberger et al. [50] contended that it does.

Table 1 summarizes the influencing factors on panic considered in the literature. It also shows the different investigational approaches employed to validate the influencing factors.

Four distinct approaches were used to find influencing factors in the literature: questionnaires, content analytics, simulations, and transactional data (Table 2). Among these, questionnaires have been widely used as there has been an extremely limited amount of objective information about the factors. However, the need for questionnaires is reduced after pandemics, especially that of COVID-19, because much quantitative data has become available. Content analytics approaches are beneficial but need very rigorous procedures for accurate results. Content analytics primarily reveal correlations between phrases, not necessarily cause-and-effect relationships. This survey shows a lack of discussions on the relative significances of influencing factors, which can help quantitative decisions.

### 4.2. Transformation of Panic to Panic Demand and Stockpiling

This section reviews the approaches to models of the relationships between the influencing factors and panic demand/stockpiling. The models have mostly considered a small number of factors and have subjectively estimated panic demand (Table 3). This survey reviews studies that incorporated all of the following considerations in their analysis.

1. Definition of selected influencing factors: the studies defined the factors that influenced panic and panic demand.
2. Correlation between the influencing factors and panic: the studies provided an explanation of how the influencing factors were correlated with panic and their relative importance in driving panic demand.
3. Quantitative estimation of panic demand: the studies clearly outlined a procedure to estimate panic demand based on the influencing factors.

Among the influencing factors of panic in Table 1, Table 3 shows that only a few of them were considered in the quantitative analytics of panic and panic demand. As Arafat et al. (2020a) [73] discussed, almost no research has reported on the causal linkages of influencing factors on the emergence of panic and the following panic demand. The quantitative models were seldom verified except in Govindan et al. [70] and Shoukat et al. [71], who used statistical analysis to evaluate the demand. It has been demonstrated that data-driven frameworks and AI can detect demand anomalies raised by panic [74,75]; however, these models do not predict future demand by influencing factors or propose intervention plans.

### 4.3. Intervention Strategies

The goal of panic research is the mitigation of the panic’s effect on society and businesses. Panic buying is a multi-dimensional process because of its multiple aspects of influential factors, ranging from psychological and social factors to economic factors. These intricacies can make some interventions actually worsen the situation. Measures such as the announcement of a lockdown, rationing, or price limitations can exacerbate panic buying [59,76,77,78]. A holistic understanding of the interactions between interventions and their affecting stages of the SEFP is needed.

Table 4 presents a summary of the qualitative and quantitative interventions discussed in the recent literature, along with their effective stages in the SEFP (Figure 1), the related key players, and the effectiveness length of these interventions.

These interventions are summarized as follows:

1. Education: This group includes the interventions related to public/governmental education about the phenomenon to increase people’s awareness and resilience. This intervention concentrates on the mitigation of the effects of influencing factors.
2. Governmental control: As the key players, governments can impose many types of controls in various stages of the SEFP. For instance, censoring rumors and media reports is a governmental control on the influencing factors. Other controls include regulation of price and shopping times, supply monitoring, and punishment of untoward sellers.
3. Information distribution: The goal is to provide clear and reliable information to mitigate the influencing factors. Effective announcements of health guidelines by governments and clear and timely announcements of available stock by retailers are the applicable interventions in this area.
4. Sustainable behavior: These interventions are based on the philanthropy of people. These solutions try to raise people’s respect toward others rather than diminish the effect of influencing factors. Even when people are in panic, for example, they can help others to meet their needs. Promoting sustainable consumption behaviors (SCBs) and the willingness to limit demand are two examples.
5. Rationing: Found under different names such as “quota policy,” “limiting sales per person,” “purchase limitation for buyers,” and “uniform rationing,” this group tries to avoid shortages and long queues in stores. Rationing can be used as a short-term palliative strategy as it does not consider the root causes of panic but just tries to ensure a better supply [68,76].
6. Subsidizing: Subsidies can be employed by governments or business parties. This intervention concentrates on the supply side and tries to promote more production with fewer price hikes or less sale fluctuation.
7. Supply resilience: There are many proposed solutions to elevate the suppliers’ resilience. Assurance of stocks, development of governmental storage and distribution systems, concurrent location and routing modeling, development of backup sites and suppliers’ flexibility, product substitution, E-commerce and locally producing strategic items are some of the interventions in this group.

**Figure 2:**
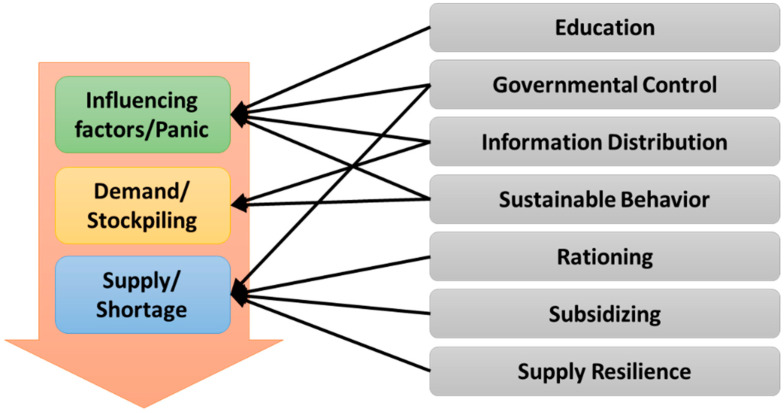
Applicable intervention strategies in different steps of the SEFP.

Each of the above interventions does not individually act on all stages of the SEFP (Figure 2), and general categorizations such as economic and psychological interventions prove to be insufficient [29]. Care in the selection of interventions provides an effective and comprehensive plan. For instance, the intervention of “directing the information flow” in the news can reduce rumors, but this intervention can diminish people’s trust if used to conceal or downplay crisis-related facts.

The literature shows some disagreements regarding the level of effectiveness among interventions and their perceived importance, e.g., social intervention is considered effective in Fu et al. [35] but least effective in Prentice et al. [78]. The literature also shows that a quantitative analysis on subsidies or supply resilience interventions, for example, is used only for the supply, but not for the other stages of the SEFP. While the promotion of sustainable consumption behaviors (SCBs) enhances mindful consumption in panic situations [51], the affected individuals may fall into lower levels of Maslow’s hierarchy of needs [87] and may not adhere to their original SCBs even with the promotion.

An action plan for the implementation of interventions needs clear definitions of the roles and responsibilities of the key stakeholders along with the timing of the interventions. Interventions and support by governments and businesses have a more significant influence on panic than those of social groups [78], and retailer interventions have only a moderating effect on panic buying [8,55]. On the other hand, improper governmental policies and flawed supply strategies are sometimes detrimental to panic situations [24]. A clear role definition becomes more important when interventions require the collaboration of multiple stakeholders. Business parties are primarily concerned about their own outcomes rather than social welfare as shown in Refs. [80,81,82,83], and sometimes, the optimal strategy for a business can even be to “wait and see” [22].

When developing and implementing intervention strategies in a panic situation, the following considerations are needed:

1. The relative magnitude of impact, aligned with the proposed SEFP.
2. The potential consequences the intervention strategies may have on the supply–demand balance.
3. The prerequisites to facilitate successful implementations.
4. The expected execution timeframes, as well as the plausible duration of the effect of the intervention.
5. The role of key stakeholders within the intervention process.

## 5. Implications and Future Research

The research team conducted an extensive literature review on panic phenomena, encompassing the examination of underlying factors, the progression from panic to panic demand, and intervention strategies. This review underscored the intricate nature of panic phenomena and their implications for both individuals and societies. The noteworthy implications of this comprehensive literature review provided concrete insights into real-world scenarios. For instance, in the context of a public health crisis, a sudden surge in panic demand for personal protective equipment (PPE) within a community serves as a pertinent case study. Our analysis not only identified contributing factors to the panic but also delineated the effectiveness of specific intervention strategies, such as education and sustainable consumption programs, in mitigating panic levels. Additionally, our review delved into the aftermath of panic situations, examining the enduring impact on long-term consumption trends. Instances of a heightened demand for medical supplies during a pandemic followed by a decreased demand in the post-crisis period illustrate the necessity of aligning supply chains with actual consumption patterns. Through the presentation of such concrete cases, this paper bridges theoretical insights with practical implications, highlighting the imperative for tailored strategies in the management and prevention of panic phenomena.

This review also identified the needs for further investigation to deepen the understanding of panic buying behavior and develop effective measures to address it. The research on the following issues will contribute to more effective crisis management, ensuring the stability of the supply chains and the wellbeing of individuals and communities during pandemics or other crises.

- A detailed understanding of influencing factors: There is a need for further exploration and agreement on the relative importance and effects of influencing factors on panic. Future research needs to identify and analyze a broader range of factors that contribute to panic, considering both internal and external factors. As summarized in the internal and external factors in 4.1, a deeper understanding of the nuances and interactions between these factors will enhance the ability to predict and mitigate panic situations. For example, to what extent will the external factors of information intensity and community preparedness affect each other and people’s internal factors such as anxiety?
- Quantitative modeling of panic demand: Future research should focus on developing quantitative models that can accurately estimate panic demand based on influencing factors. These models should go beyond simplistic representations of fluctuating demand and incorporate the respective effects of each underlying cause and the dynamics of panic buying behavior. By doing so, policymakers and businesses can make informed quantitative decisions to stabilize demand–supply cycles during panic periods.
- The effectiveness of intervention strategies: The effectiveness of intervention strategies needs to be evaluated more rigorously. By identifying the most effective strategies, policymakers can develop evidence-based measures to manage panic situations more efficiently. For example, some strategies will affect situations differently based on customer categories (e.g., gender) and product types (e.g., fresh food and staples). Also, future research should assess the impact of different interventions on the stages of the SEFP and evaluate possible side effects.
- Long-term consumption trends: To ensure the effective long-term planning of stakeholders, it is necessary to investigate the relationships between panic demand and consumption trends before, during, and after a crisis. This understanding will help determine the actual supply quantities needed for consumption. It is worth noting that different product types display distinct usage patterns. For instance, as discussed in Engstrom et al. [59], there was a surge in demand for diabetes medication during a panic situation. Because the consumption remained unchanged, after the panic, new demand decreased as people consumed their stockpiled medicines. In contrast, masks and sanitizers experienced an increase in both demand and consumption during and after a panic situation.
- Psychological factors and tolerance building: Future research should explore strategies to raise individuals’ tolerance and avoid panic buying. While it is not possible to eliminate the influencing factors, people can better tolerate the situation with a deeper understanding of the psychological factors and sustainable consumption behaviors.
- These prospective investigations will deepen our understanding of panic, refine quantitative models, rigorously evaluate interventions, analyze long-term consumption trends, and explore strategies for enhancing individual tolerance to mitigate consumers’ panic buying behavior more effectively.

## Figures and Tables

**Table 1:** Influencing factors of panic.

Ref.	Influencing Factors	Investigation Approach	Ref.	Influencing Factors	Investigation Approach
[8]	- Affective response - Cognitive response - Governmental intervention - Community preparedness	Text Analytics	[41]	- Empty shelves - Infection/health - Distrust - Background rate	Survey
[12]	- Information quality - Anxiety	Questionnaire	[51]	- Intolerance of uncertainty - Expected quality of life	Survey
[14]	- Fear of illness - Empty shelves - Price increases - Social inclination to buy	Interview	[47]	- Food at hand - Infection possibility - Bad mood - Herd psychology	Survey
[15]	- Act based on others	Simulation	[52]	- Amount of shared info.	Survey
[25]	- Death/injury/damage - Loss of job/income - Isolation - Displacement - Peer behavior - Governmental intervention - Supply disruption - Price changes - Shortages	Text Analytics	[53]	- Perceived severity - Perceived susceptibility - Social influence - Social norms - Perceived scarcity - Affective response - Perceived lack of control - Available information	Survey
[33]	- Supply disruption - Demographics - Intolerability - Emotional contagion	Interview	[54]	- Effective information release - Sufficiency of supplies - Others’ behavior people - Conformity of community	Simulation
[34]	- Shortages - Delivery limitations - Payment/credit limit	Interview	[49]	- Attitude - Subjective norm - Online news	Survey
[48]	- Perceived scarcity - Perceived severity - Normative social influence - Social trust - Observational learning	Survey	[46]	- Anticipated regret - Perceived scarcity - Perceived susceptibility - Perceived severity - Outcome expectation - Cues to action - Self-efficacy	Survey
[50]	- Attitude - Fear of unavailability - Subjective norm	Questionnaire	[55]	- Government measures - Media - Peer influence	Questionnaire
[56]	- Sense of scarcity - High demand for product - Importance of the product - Price hikes	Text Analytics	[57]	- Fear of lockdown - Peer buying - Limited supply - Governmental control	Survey
[58]	- Exposure to information - Anxiety	Survey	[59]	- Death - Lockdowns	Business Data
[60]	- Limited quantity scarcity - Limited time scarcity - Social media usage	Survey	[61]	- Thinking style - Situational ambiguity - Information overload	Survey
[62]	- eWOM	Questionnaire	[63]	- Anxiety	Text Analytics
[64]	- Social media	Interview	[65]	- Data Availability	Business Data

**Table 2:** Different investigational approaches and their popularity to find the influencing factors.

Investigation Approach	Coverage Area	No. of Papers
Survey	Online/offline questionnaires and interviews with consumers or business parties	20
Content Analytics	Text mining and research analytics in reports and social media, especially Twitter	4
Simulation	Data generated by algorithms	2
Transactional Data	Real data of businesses or governments	2

**Table 3:** Quantitative studies of factors influencing panic demand.

Ref.	Influencing Factors	Model	Key Points
[13]	- Information intensity (rumor)	Simulation	- Rumor is considered an indirect factor, which changes the perceived value of an item and causes panic.
[15]	- Susceptibility - Information intensity - Regulation	Clustering and Simulation	- It shows how factors motivated people to buy more, but their demands were not discussed. - Real data were not used and just a simulation was used.
[38]	- Information intensity (rumor) - Shortage	Simulation	- Shortage was considered a fact without being imported into the model. - Panic was considered in the [0, 1] range.
[54]	- Information intensity (rumor) - Statistics - Neighboring effect	Simulation	- Relative importance of factors was obtained by simulation. - Panic was considered in the [0, 1] range. - It was not clearly explained how panic transformed to demand.
[66]	- Income	Statistical Analytics	- It correlated the income to purchase amounts. But the correlation did not necessarily show the cause–effect relationships.
[67]	- Information overload	Simulation	- A sigmoid function was used to estimate panic. - Panic was considered in the [0, 1] range. - Distance determined the intensity of neighbors’ panic. - Panic was also related to previous panic.
[68]	- Regulation - Statistics - Information intensity (rumor) - Neighboring effect	Simulation	- Panic intention was defined based on the sigmoid function. - Demand was not discussed.
[69]	- Regulation - Statistics - Information intensity (rumor) - Neighboring effect	Statistical Analytics	- Data analytics was based on survey data rather than on transactional data.
[70]	- Anxiety	Statistical Analytics	- Just one factor was analyzed. - Real data were used to evaluate the model performance.
[71]	- Community preparedness	Statistical Analytics	- Just one factor was analyzed. - Real data were used to evaluate the model’s performance. - Demand evaluation was not considered.
[72]	- Neighboring effect	Mathematical Model	- Just one factor was considered. - Parameter estimation was not considered.

**Table 4:** Applicable intervention strategies in panic-related research.

Ref.	Interventions		Key Player ^1^	Area of Effect ^2^	Effective-ness ^3^	Dec. Making Method
	Title	Group				
[13]	- Real-time monitoring of the rumors and promoting refuting them	Information Distribution	GOV	Influencing Factors	S	Mathematical Modeling
	- Effective management of emergency supplies	Rationing	GOV	Supply	L	
[19]	- Raise awareness level about the phenomenon	Education	GOV	General	L	Survey
[21]	- Increase robustness of supply by considering more backups	Supply Resilience	BUS	Supply	L	Mathematical Modeling
[32]	- Remove profit motives of purchasing in costing models	Subsidizing	BUS	General	N	Contextual Discussion
	- Develop government storage and distribution systems	Governmental Control	GOV	Supply	N	
	- Develop and enforce regulations	Governmental Control	GOV	General	N	
	- Reduce US dependence on import of strategic products	Governmental Control	GOV	Supply	N	
[35]	- Supply monitoring	Supply Resilience	BUS	Supply	N	Mathematical Modeling
	- Information review and guidance	Information Distribution	GOV/BUS	General	N	
[44]	- Improved routing and location-allocation models with low-resources setting	Supply Resilience	BUS	Supply	L	Summary
[47]	- Providing clear and timely information to stockpile	Information Distribution	GOV/BUS	Demand/ Stockpiling	N	-
	- Public education	Education	GOV	General	N	
[56]	- Raising awareness	Education	GOV	Influencing Factors	N	Summary
	- Dissemination of stock status to the general population	Information Distribution	GOV/BUS	Influencing Factors	N	
	- Assurance of stocks	Supply Resilience	BUS	Supply	N	
	- Rationing and selling lower-priced goods	Rationing	GOV	Supply	N	
	- Setting monitoring team to punish maleficent sellers	Governmental Control	GOV	Supply	N	
[51]	- Promote sustainable consumption behaviors (SCBs)	Sustainable Behavior	GOV	Demand/ Stockpiling	L	Survey
[58]	- Effective official information distribution	Information Distribution	GOV	Influencing Factors	S	Survey
	- Increase people’s resilience	Sustainable Behavior	GOV	General	L	
[63]	- Effective government announcements	Information Distribution	GOV	Influencing Factors	S	Statistical Analytics
[65]	- Effective information sharing of available products	Information Distribution	GOV/BUS	Influencing Factors	S	Survey
[67]	- Implementing a quota policy	Rationing	GOV	Supply	N	Mathematical Modeling
	- Uniform rationing	Rationing	GOV	Supply	N	
[68]	- Implementing a quota policy	Rationing	GOV	Supply	N	Simulation
	- Rationing uniformly	Rationing	GOV	Supply	N	
	- Control media reports	Information Distribution	GOV	Influencing Factors	N	
[71]	- Increase people’s awareness about self-isolation	Education	GOV	Demand/ Stockpiling	S	Real Data Analytics
[76]	- Price regulation for suppliers	Subsidizing	GOV	Supply	S	Mathematical Modeling
	- Purchase limitation for buyers (not effective to prevent shortages)	Rationing	GOV/BUS	Demand/ Stockpiling	S	
[79]	- Different forms of messaging (by those who are not community leaders)	Information Distribution	GOV	General	N	Contextual Discussion
[80]	- Product substitution based on customer segmentation	Supply Resilience	BUS	Supply	S	Mathematical Modeling
[81]	- Increased flexibility in suppliers causes more profitability	Supply Resilience	BUS	Supply	L	Mathematical Modeling
[82]	- Concurrent location and routing modeling	Supply Resilience	BUS	Supply	L	Mathematical Modeling
[83]	- Subsidizing the supply and customer purchases	Subsidizing	BUS	Demand/ Stockpiling/Supply	S	Mathematical Modeling
[84]	- Promoting willingness to limit demand relying on trusted change agents	Sustainable Behavior	GOV	Demand/ Stockpiling	L	Survey
[85]	- Effective government announcements	Information Distribution	GOV	Influencing Factors	S	Survey
[86]	- Limiting sales per person to control the excess demand	Rationing	GOV	Demand/ Stockpiling	S	Simulation

^1^ BUS: business, GOV: government. ^2^ General: this refers to interventions that will affect multiple areas in the SEFP. ^3^ S: short-term, L: long-term, N: no information is available.

## Data Availability

Data sharing is not applicable for Review type manuscripts.
